# Comparing the adverse effects of ketamine and esketamine between genders using FAERS data

**DOI:** 10.3389/fphar.2024.1329436

**Published:** 2024-07-12

**Authors:** Xinxia Yang, Dongdong Chen

**Affiliations:** The Department of Anesthesiology, Ningbo Medical Center Lihuili Hospital, Ningbo, China

**Keywords:** esketamine, ketamine, gender difference, signal mining, adverse drug events

## Abstract

**Background:**

Ketamine was developed as an anesthetic. Esketamine is the isolated S-enantiomer of racemic ketamine. They provide new avenues for the treatment of depression, especially treatment-resistant depression. Considering differences in the pharmacokinetics and hormonal status of ketamine in patients of different genders, sex-based differences in esketamine adverse drug events (ADE) may also be observed. This study presents data mining and safety analysis of adverse events of ketamine and esketamine between genders, promoting the individualization of clinical practice.

**Methods:**

Adverse drug reactions to ketamine and esketamine reported between the first quarter of 2004 and the second quarter of 2023 in the U.S. Food and Drug Administration on Adverse Event Reporting System (FAERS) were extracted. Thereafter, the reporting odds ratio (ROR) with 95% confidence interval (CI) was calculated.

**Results:**

A total of 2907 female reports and 1634 male reports on esketamine were included in the analysis. ROR mining showed that completed suicide, decreased therapeutic product effects, urinary retention, and hypertension were common in men. Additionally, 552 female and 653 male ketamine reports were recorded. ROR mining revealed that toxicity to various agents, bradycardia, cystitis and agitation, were more likely to occur in men, whereas women were more likely to develop suicidal ideation, increased transaminase levels, sclerosing cholangitis, and sterile pyuria.

**Conclusion:**

The adverse events of esketamine and ketamine differ across genders, which should be considered in clinical practice to provide individualized treatment.

## 1 Introduction

Ketamine was originally developed as an anesthetic and approved by the Food and Drug Administration (FDA) in 1970. Esketamine, a novel antidepressant that is the isolated S-enantiomer of racemic ketamine, was approved for treatment in March 2019. Esketamine’s efficacy and safety in treatment-resistant depression were evaluated in three 4-week, placebo-controlled, parallel-group studies and one longer-term randomized withdrawal study. To mitigate the risk of serious adverse outcomes resulting from sedation, dissociation, and abuse and misuse, while providing access to this effective treatment for treatment-resistant depression, the FDA approved esketamine with a Risk Evaluation and Mitigation Strategy (REMS) (Kim et al., 2019). Esketamine has good efficacy and tolerability even in more difficult-to-treat populations, such as those with comorbid substance use (Chiappini et al., 2023). A real-world study compared the antidepressant effects and tolerability of ketamine and esketamine, with data that seems to favor the nasal spray formulation in terms of Treatment Emergent Side Effects. The results indicate that, ketamine showed a higher short-term antidepressant effect, whereas esketamine exhibited lower side effects. Both were generally well tolerated (d'Andrea et al., 2024). In a phase 3b trial involving patients with treatment-resistant depression, esketamine was superior to quetiapine with respect to remission at week 8. The most common adverse reactions in the esketamine group include dizziness, nausea, drowsiness, etc (Reif et al., 2023). Ketamine is safe as an anesthetic, analgesic, and antidepressant. When ketamine is used as an entertainment drug, it can lead to serious consequences such as cognitive impairment, mental illness, bladder and nasal mucosal damage, and so on (Cheng et al., 2018). Treatment-emergent adverse events with ketamine and esketamine in major depression may be categorized as psychiatric (e.g., dissociation, psychotomimetic), neurologic/cognitive, hemodynamic, genitourinary, and abuse liability (Short et al., 2018). There is currently no research on gender differences between ketamine and esketamine. Considering the differences in the pharmacokinetics and hormonal status of ketamine in patients of different genders (Zarate et al., 2012; Chen et al., 2020), differences in esketamine regarding adverse drug events (ADE) based on sex may also be observed. Therefore, this study aimed to analyze differences in the ADE of esketamine and ketamine based on gender. Relevant data were extracted from the U.S. Food and Drug Administration Adverse Event Reporting System (FAERS). Signal mining and evaluation of the differences in ADE of esketamine and ketamine between genders were conducted based on data-mining algorithm and statistical testing (Park et al., 2020). Drug vigilance and safety monitoring were also performed. This study aimed to provide decision support for patients of different genders in the treatment plan and provide a reference for clinically rational medication.

## 2 Materials and methods

The data for this study were sourced from the FAERS database, which is publicly available since 2004 (updated quarterly). This study extracted all ASCII data packages from the first quarter of 2004 to the second quarter of 2023 and imported them into SAS9.4 software for data cleaning and analysis. Then, according to CASEID, FDA_ DT and PRIMARYID remove duplicate information. The FAERS database includes spontaneous safety reports and post-market clinical research reports related to medications used both in the US and overseas. The latest version of the Medical Dictionary for Regulatory Activities (MedDRA) was used to correct the preferred term (PTs) names in the FAERS database, as PT from MedDRA was used to record adverse reactions in the FAERS database. The MedDRA dictionary is updated annually in March and September, and each update involves adjustments to the PT hierarchy and changes in the system organ class (SOC). Therefore, SOC and PT were obtained from the latest version of the MedDRA. Next, with “ESKETAMINE,” “SPRAVATO,” “KETAMINE,” and “KETALAR” as keywords, the database was further defined with “PS (primary suspect drug)” to exclude non-primary suspected drugs.

The disproportionality method mainly includes frequency and Bayesian methods (Jokinen et al., 2018). Frequency methods include reporting odds ratio (ROR) and medicines and healthcare products regulatory agency (MHRA). MHRA evaluates adverse event based on three indicators: proportional reporting ratio (PRR), *X* ^*2*^, and number of reports. Bayesian methods include the Bayesian confidence propagation neural network (BCPNN) and multi-item gamma Poisson shrinker (MGPS) methods. In this study, these four methods were adopted for signal mining (Li et al., 2023). The detailed contents of the four algorithms are provided in the Supplementary File S1. Signals that meet three or more algorithms are considered potential ADE signals related to ketamine or esketamine. The frequency method is simple and sensitive, but has low stability and high false positives (Chen et al., 2022). When the number of reports is reduced, Bayesian method can effectively avoid false positives, but the calculation is complex and the signal detection time is relatively delayed (He et al., 2021; Li et al., 2023). Multiple algorithms can be used to mine high-quality signals, providing a basis for clinical drug safety and further research.

To identify gender differences in ADEs, the obtained potential signals were further analyzed at the PT levels and classified into different SOCs to better outline the signals. The gender data in the 2 × 2 contingency table along with a modified ROR signal mining method was employed as shown in Table 1 (Li et al., 2023).

**TABLE 1:** A 2 × 2 contingency table for disproportionality analysis of the gender difference in ADEs.

Gender	Adverse event	Not adverse event	Total
Female	a	b	a+b
Male	c	d	c+d
Total	a+c	b+d	a+c+b+d

ROR=(a/c)/(b/d), 95% confidence interval (CI) = $e^{\ln\left(ROR\right)\pm 1.96\sqrt{\frac{1}{a}+\frac{1}{b}+\frac{1}{c}+\frac{1}{d}}}$ . Signal detection standards: ① Reports a≥3; ② When ROR>1 and 95% CI>1, women are more likely than men to report a specific ADE, with a stronger correlation observed at higher values. When ROR was <1 and 95% CI was <1, men were more likely than women to report a specific ADE, and the higher the value, the stronger the correlation.

## 3 Results

### 3.1 General characteristics

Based on the extracted basic information from ADE reports, a total of 5541 reports for esketamine were obtained, including 2907 reports for women, 1634 reports for men, and 1000 reports for unknown sex. The total number of reports for ketamine ADE was 1646, with 552 reports for women, 653 reports for men, and 441 reports for unknown sex. The clinical outcomes, age distribution, reporting population of patients and country of the reporter are shown in Table 2. More deaths were reported in the men. The country with the most reports is mainly the United States of America.

**TABLE 2:** Basic information on the ADE reports of esketamine and ketamine.

Items	Ketamine			Esketamine		
	Female	Male	Unknow	Female	Male	Unknow
Outcome						
Life-Threatening	92 (1.66)	41 (0.74)	4 (0.07)	61 (3.71)	59 (3.58)	12 (0.73)
Hospitalization	714 (12.89)	356 (6.42)	44 (0.79)	189 (11.48)	203 (12.33)	134 (8.14)
Death	100 (1.80)	114 (2.06)	20 (0.36)	57 (3.46)	120 (7.29)	41 (2.49)
Disability	24 (0.43)	4 (0.07)	1 (0.02)	15 (0.91)	17 (1.03)	0
Required intervention to prevent permanent impairment/Damage	5 (0.09)	1 (0.02)	0	9 (0.55)	14 (0.85)	2 (0.12)
Other	1150 (20.75)	649 (11.71)	140 (2.53)	345 (20.96)	395 (24)	264 (16.04)
Reporter						
Physician	1043 (18.82)	573 (10.34)	344 (6.21)	211 (12.82)	305 (18.53)	104 (6.32)
Consumer	995 (17.96)	588 (10.61)	171 (3.09)	68 (4.13)	46 (2.79)	31 (1.88)
Pharmacist	809 (14.60)	443 (7.99)	416 (7.51)	121 (7.35)	154 (9.36)	86 (5.22)
Other health-professional	59 (1.06)	29 (0.52)	69 (1.25)	121 (7.35)	118 (7.17)	200 (12.15)
Missing	1 (0.02)	1 (0.02)	0	30 (1.82)	29 (1.76)	18 (1.09)
Lawyer	0	0	0	1 (0.06)	1 (0.06)	2 (0.12)
Age						
<18	42 (0.76)	9 (0.16)	0	65 (3.95)	93 (5.65)	18 (1.09)
≥18,<45	780 (14.08)	455 (8.21)	19 (0.34)	263 (15.98)	354 (21.51)	3 (0.18)
≥45,<65	857 (15.47)	453 (8.18)	23 (0.42)	114 (6.93)	97 (5.89)	6 (0.36)
≥65,<75	199 (3.59)	105 (1.89)	8 (0.14)	33 (2.00)	30 (1.82)	0
≥75	45 (0.81)	36 (0.65)	1 (0.02)	12 (0.73)	10 (0.61)	0
Missing	984 (17.76)	576 (10.40)	949 (17.13)	65 (3.95)	69 (4.19)	414 (25.15)
Country of the reporter (top five)						
United States of America	317 (57.43)	331 (50.69)	219 (49.66)	2260 (77.74)	1275 (78.03)	900 (90.00)
China	72 (12.86)	100 (15.31)	136 (30.84)	20 (0.69)	2 (0.12)	10 (1.0)
United Kingdom	53 (9.60)	88 (13.48)	37 (8.39)	8 (0.28)	1 (0.06)	0
France	12 (2.17)	8 (1.23)	6 (1.36)	121 (4.16)	56 (3.43)	7 (0.70)
Brazil	0	2 (0.31)	0 (0.00)	66 (2.27)	39 (2.39)	8 (0.80)

### 3.2 Identification of potential ADE signals

Through signal mining of esketamine, 161 PT signals reached the threshold. Excluding signals unrelated to drugs or disease progression, such as product issues, social circumstances, and surgical and medical procedures, 151 PT signals were ultimately obtained from 13 system organ classes (SOCs), as shown in Supplementary Table S1. The top three SOC level reports were for psychiatric disorders (n = 3916), nervous system disorders (n = 1990), and general disorders and administration site conditions (n = 1699). At the PT level, the most frequent reports were dissociation, sedation, and suicidal ideation, consistent with the warning items in drug instructions. The top 50 ADEs related to esketamine, in terms of ROR values, are shown in Table 3. This study discovered new potential ADE signals with clinical value such as flashbacks, tachyphylaxis, conversion disorders, essential tremors, and agoraphobia. These signals are not mentioned in the drug label.

**TABLE 3:** Top 50 Preferred Terms of ADEs related to Esketamine in the ROR values.

Preferred terms (PTs)	Cases	ROR	PRR	*X* ^*2*^	EBGM05	IC 025	Unexpected signal
Dissociation	921	1923.92	1779.35	788,599.35	780.03	0.00	—
Dissociative disorder	37	354.76	353.70	10,720.23	204.34	1.16	—
Sedation	593	155.72	148.23	79,613.00	124.91	0.20	—
Morbid thoughts	13	86.78	86.69	1046.28	47.18	1.07	—
Flashback	4	61.84	61.82	230.73	21.97	1.74	Yes
Derealisation	17	50.36	50.29	797.11	30.13	2.04	—
Euphoric mood	75	47.19	46.91	3277.41	36.26	0.88	—
Tachyphylaxis	3	46.82	46.80	130.78	14.45	8.68	Yes
Psychogenic seizure	7	45.60	45.58	297.01	20.94	0.87	—
Autoscopy	7	42.90	42.88	279.06	19.74	0.21	—
Drug monitoring procedure incorrectly performed	7	41.66	41.64	270.84	19.19	3.21	—
Suicidal ideation	536	41.58	39.80	19,819.98	35.63	0.32	—
Illusion	11	40.64	40.61	414.76	21.80	2.52	—
Major depression	50	39.63	39.47	1831.23	29.12	0.58	—
Depressive symptom	21	33.07	33.01	639.17	21.02	0.25	—
Feeling drunk	33	32.69	32.60	991.39	22.66	0.66	—
Feeling of relaxation	3	32.22	32.22	89.01	10.09	4.57	—
Negative thoughts	11	25.72	25.69	257.08	13.95	0.41	—
Nasal discomfort	31	24.95	24.89	700.27	17.20	1.08	—
Flat affect	6	24.45	24.44	132.92	10.76	0.25	—
Dysphoria	11	22.26	22.24	220.17	12.11	2.49	—
Conversion disorder	5	21.88	21.88	98.31	8.94	2.18	Yes
Alcohol poisoning	7	20.00	19.99	124.80	9.38	0.20	—
Self-injurious ideation	12	18.92	18.90	201.15	10.58	1.51	—
Suicide attempt	171	17.45	17.22	2588.17	14.65	0.19	—
Panic attack	96	16.70	16.57	1391.62	13.42	0.43	—
Essential tremor	3	16.49	16.48	43.20	5.24	6.67	Yes
Panic disorder	10	15.58	15.57	135.10	8.28	1.63	—
Feeling of despair	12	15.05	15.03	155.80	8.44	0.37	—
Hyperacusis	9	14.49	14.48	111.95	7.45	2.24	—
Agoraphobia	3	14.34	14.34	36.91	4.56	2.80	Yes
Bipolar I disorder	4	14.20	14.20	48.65	5.26	5.05	—
Logorrhoea	6	13.94	13.94	71.45	6.19	1.64	—
Inappropriate affect	3	13.34	13.34	33.96	4.25	0.91	—
Depression suicidal	9	13.02	13.01	99.01	6.70	0.74	—
Disturbance in social behaviour	6	12.81	12.80	64.79	5.69	1.15	—
Fear of death	4	12.53	12.53	42.12	4.65	2.39	—
Device dispensing error	4	12.30	12.30	41.20	4.57	4.06	—
Motion sickness	4	11.99	11.98	39.98	4.45	3.12	—
Post-traumatic stress disorder	18	11.57	11.55	172.30	7.22	1.81	—
Cystitis interstitial	3	11.41	11.41	28.28	3.64	3.05	—
Hypomania	5	11.35	11.34	46.83	4.68	3.74	—
Disinhibition	3	11.28	11.28	27.90	3.60	4.51	—
Depression	380	10.87	10.57	3279.63	9.48	0.69	—
Bladder spasm	3	10.86	10.86	26.67	3.47	0.44	—
Staring	3	10.32	10.31	25.08	3.30	1.75	—
Somatic symptom disorder	4	10.18	10.18	32.90	3.79	3.56	—
Sensory disturbance	23	10.16	10.14	188.44	6.69	0.88	—
Therapeutic product effect increased	5	9.62	9.61	38.37	3.97	3.53	—
Hypoaesthesia oral	23	9.53	9.52	174.32	6.28	0.67	—

Note: ROR, reporting odds ratio; PRR, proportional reporting ratio; *X* ^*2*^, chi-squared; EBGM, empirical Bayesian geometric mean; IC, information component.

Ketamine yielded 221 PT signals. Excluding signals unrelated to the drug or disease progression, 215 PT signals were mapped to 16 SOCs, as shown in Supplementary Table S2. The top three frequencies of SOC level reports were psychiatric disorders (n = 1018); renal and urinary disorders (n = 1990); and injury, poisoning, and procedural complications (n = 639). At the PT level, the most frequent reports were drug abuse, off-label use, and hydronephrosis, consistent with the symptoms of drug abuse. The top 50 PTs of ADEs related to ketamine in terms of ROR values are shown in Table 4.

**TABLE 4:** Top 50 Preferred Terms of ADEs related to Ketamine in the ROR values.

Preferred terms (PTs)	Cases	ROR	PRR	*X* ^*2*^	EBGM 05	IC 025	Unexpected signal
Ureteral polyp	18	19,136.17	19,077.04	105,646.43	2551.58	3.38	—
Cystitis ulcerative	34	13,807.98	13,727.39	178,180.76	3040.75	4.50	—
Biopsy bladder abnormal	11	11,680.25	11,658.19	53,986.59	1973.94	2.53	—
Reduced bladder capacity	31	6445.01	6410.71	113,131.74	2288.25	4.37	—
Sterile pyuria	15	3863.89	3853.95	39,723.25	1438.55	3.16	—
Contracted bladder	36	2625.04	2608.83	71,764.62	1372.05	4.65	—
Lower urinary tract symptoms	42	1749.70	1737.10	60,482.83	1033.53	4.91	—
Ureteritis	14	1166.54	1163.74	14,301.48	585.04	3.09	—
Bladder necrosis	5	800.56	799.88	3645.48	292.10	1.34	—
Bladder hypertrophy	21	627.00	624.75	12,180.27	373.32	3.77	—
Biliary dilatation	61	450.96	446.25	25,744.85	327.32	5.38	—
Biliary tract disorder	54	420.49	416.61	21,340.74	301.83	5.20	—
Pyonephrosis	3	397.64	397.44	1133.21	119.25	0.50	—
Bladder neck obstruction	4	317.18	316.96	1214.46	112.59	0.98	—
Ureteral disorder	13	315.62	314.92	3922.38	174.47	2.96	—
Ureteric stenosis	23	305.43	304.23	6710.61	193.62	3.88	—
Urogenital fistula	5	305.25	304.99	1462.36	120.60	1.36	—
Dissociative disorder	14	241.84	241.26	3257.10	137.84	3.07	—
Ureteric injury	3	240.09	239.96	694.25	74.06	0.51	—
Hydronephrosis	153	222.31	216.50	32,005.32	179.45	6.24	—
Waxy flexibility	4	216.17	216.02	834.82	78.07	0.98	—
Intra-abdominal pressure increased	3	215.67	215.56	624.81	66.82	0.51	—
Hydroureter	7	211.47	211.21	1428.93	97.32	1.92	—
Urge incontinence	16	192.41	191.88	2970.97	114.25	3.26	—
Urinary tract disorder	74	183.82	181.49	13,005.47	140.95	5.39	—
Airway complication of anaesthesia	4	172.27	172.16	667.10	62.69	0.98	—
Cystitis interstitial	26	167.49	166.75	4201.19	110.85	3.98	—
Biliary cyst	3	153.31	153.23	445.65	48.04	0.51	—
Ureteric obstruction	19	141.27	140.82	2594.63	87.97	3.48	—
Renal papillary necrosis	3	136.09	136.02	395.74	42.78	0.51	—
Pyuria	13	133.09	132.80	1674.29	75.56	2.89	—
Diabetes insipidus	28	126.75	126.14	3425.43	85.52	4.02	—
Abdominal compartment syndrome	7	121.52	121.37	823.87	56.73	1.89	—
Metaplasia	6	116.53	116.41	677.23	51.29	1.63	—
Cystitis noninfective	14	115.86	115.58	1568.90	67.26	2.99	—
Suprapubic pain	5	101.02	100.94	488.92	41.29	1.33	—
Accidental death	18	95.32	95.03	1656.20	59.02	3.33	—
Intensive care unit acquired weakness	4	94.01	93.95	363.80	34.68	0.96	—
Hepatobiliary disease	5	93.25	93.17	450.96	38.17	1.32	—
Laryngospasm	25	92.79	92.40	2236.02	61.59	3.78	—
Delayed recovery from anaesthesia	8	91.79	91.66	709.72	45.16	2.08	—
Biliary sepsis	4	90.02	89.96	348.19	33.23	0.96	—
Dose calculation error	4	84.84	84.79	327.92	31.35	0.96	—
Hypertonic bladder	21	80.93	80.64	1636.21	51.94	3.50	—
Cholangitis sclerosing	14	80.23	80.04	1082.57	46.82	2.92	—
Vesicoureteric reflux	3	71.09	71.05	205.47	22.61	0.49	—
Pneumatosis	3	70.89	70.85	204.89	22.55	0.49	—
Necrosis ischaemic	3	70.69	70.66	204.31	22.49	0.49	—
Propofol infusion syndrome	4	61.15	61.11	234.81	22.69	0.93	—
Maternal exposure during delivery	4	61.04	61.00	234.37	22.64	0.93	—

Note: ROR, reporting odds ratio; PRR, proportional reporting ratio; *X* ^*2*^, chi-squared; EBGM, empirical Bayesian geometric mean; IC, information component.

### 3.3 Signal detection results

At the PT level, the signal detection results for esketamine showed that the high-risk male signals included completed suicide, decreased therapeutic product effect, urinary retention, and hypertension (Table 5). The signal detection results for ketamine revealed that toxicity to various agents, bradycardia, cystitis, and agitation, were more likely to occur in men, whereas women were more likely to develop suicidal ideation, increased transaminase levels, sclerosing cholangitis, and sterile pyuria (Table 6).

**TABLE 5:** Gender differences in risk signal detection for esketamine.

SOC/PT	Female/Male	ROR (95%CI)
**Renal and urinary disorders**		
Urinary retention	4/8	0.26 (0.08, 0.85)
**General disorders and administration site conditions**		
Therapeutic product effect decreased	22/26	0.43 (0.25, 0.77)
**Vascular disorders**		
Hypertension	124/85	0.75 (0.56, 0.99)
**Cardiac disorders**		
**Psychiatric disorders**		
Completed suicide	38/45	0.43 (0.28, 0.67)

Note: SOC, system organ class; PT, preferred term; ROR, reporting odds ratio; CI, confidence interval.

**TABLE 6:** Gender differences in risk signal detection for ketamine.

SOC/PT	Female/Male	ROR (95%CI)
**Psychiatric disorders**		
Suicidal ideation	18/4	5.21 (1.76,15.41)
Agitation	4/20	0.23 (0.08,0.67)
**Renal and urinary disorders**		
Sterile pyuria	12/3	4.62 (1.30,16.39)
**Hepatobiliary disorders**		
Cholangitis sclerosing	11/3	4.23 (1.18,15.19)
**Investigation**		
Transaminases increased	11/3	4.23 (1.18,15.19)
**Injury, poisoning and procedural complications**		
Toxicity to various agents	14/41	0.39 (0.21,0.71)
**Cardiac disorders**		
Bradycardia	10/26	0.44 (0.21,0.91)
**Infections and infestations**		
Cystitis	22/47	0.53 (0.32,0.89)

Note: SOC, system organ class; PT, preferred term; ROR, reporting odds ratio; CI, confidence interval.

### 3.4 Visualization of signal results

**FIGURE 1:**
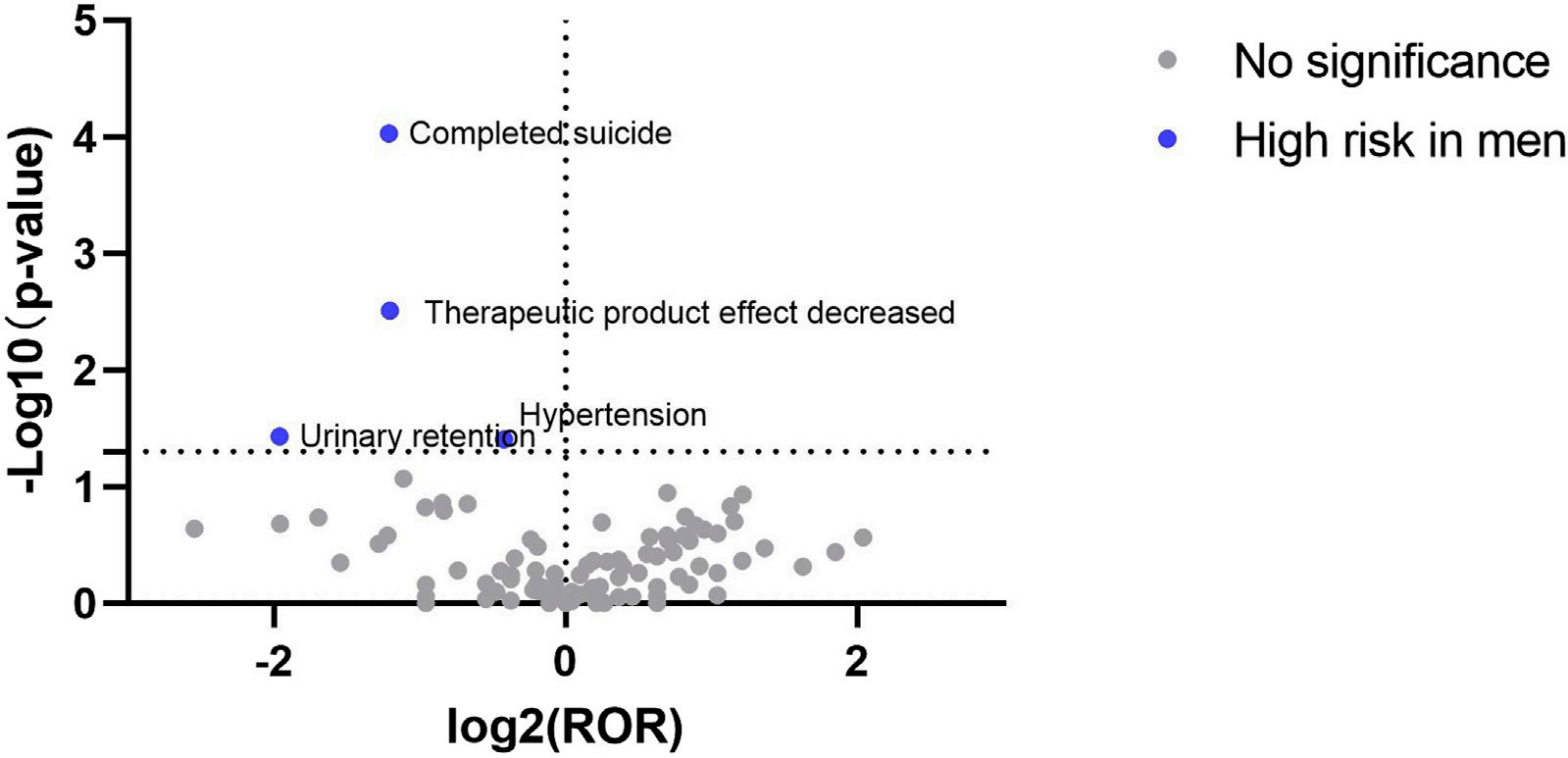
Volcanic map of gender difference risk signal for esketamine.

**FIGURE 2:**
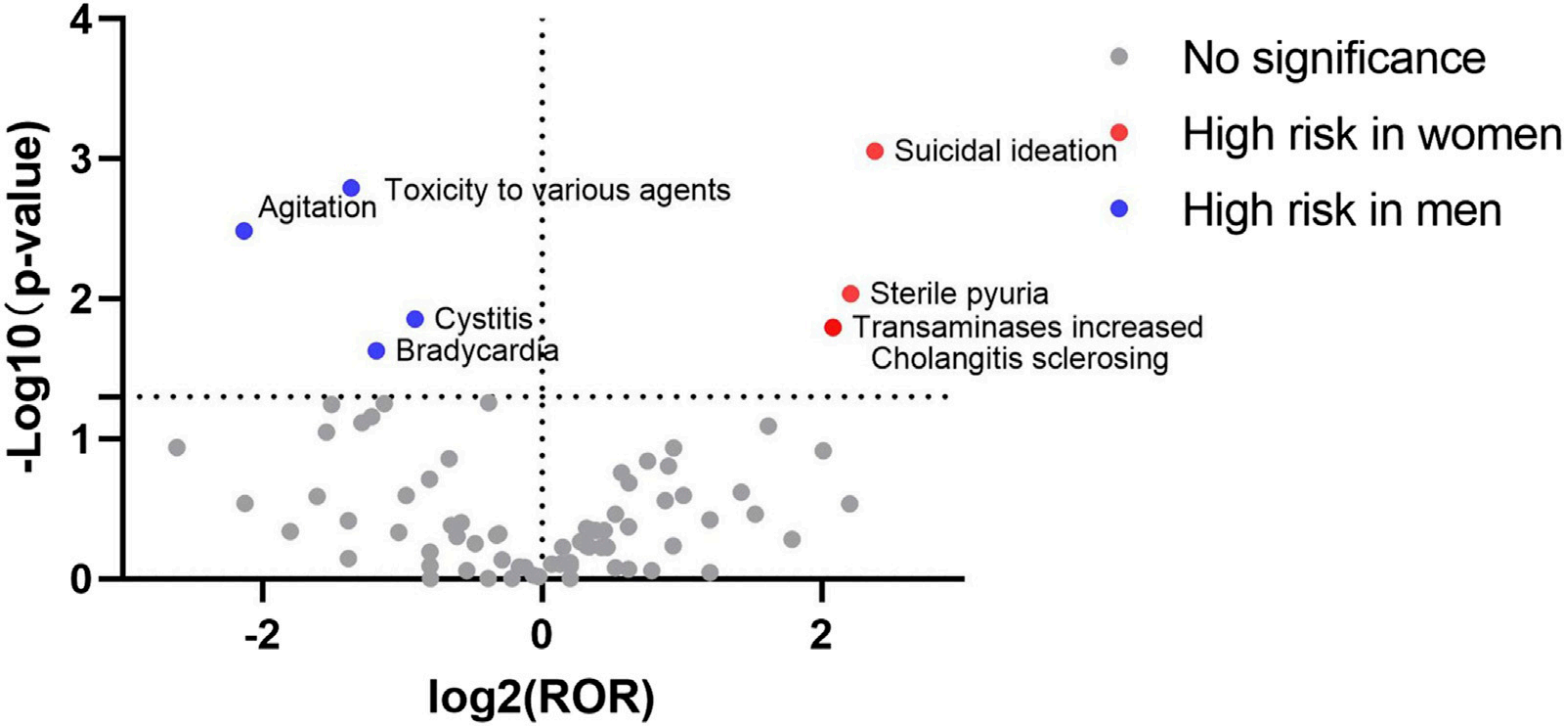
Volcanic map of gender difference risk signal for ketamine.

The signal results were visualized to analyze the gender differences in the ADE signal mining results between esketamine and ketamine. A “volcano plot” was constructed to visualize the signals. The vertical axis of the volcano plot is marked with the log_10_ *p*-value and the horizontal axis is marked with the log_2_ROR value. The results are shown in Figures 1, 2, where each point represents an adverse event. The red dots represent potential ADE signals for female patients and the blue dots represent potential ADE signals for male patients. ADE signals with significant log_2_ROR and log_10_ *p* values are shown in Figures 1, 2.

## 4 Discussion

The FAERS database has been publicly available since 2004 with quarterly updates and a significant amount of data. This can effectively support post-market safety risk monitoring and drug analysis. By mining ADE data from the FAERS database, this study revealed that men receiving esketamine had a higher risk of completed suicide, decreased therapeutic product effect, urinary retention, and hypertension. Furthermore, ketamine ADE reports indicated that males were at a higher risk of toxicity to various agents, bradycardia, cystitis, and agitation, whereas females were more likely to develop suicidal ideation, increased transaminase levels, sclerosing cholangitis, and sterile pyuria.

Through comprehensive pharmacovigilance analysis, 151 PT positive signals were associated with esketamine (Supplementary Table S1), and 215 PT positive signals were associated with ketamine (Supplementary Table S2), almost covering the information provided in the instruction manual. This indicates that the data mining strategy used in this study is feasible for identifying potential ADE signals. In addition, this study found some important ADEs that were not mentioned in the esketamine instruction manual, including flashbacks, tachyphylaxis, conversion disorders, essential tremors, and agoraphobia. The PT signals were strong. Randomized controlled trials are the gold standard for determining therapeutic effects, but have some shortcomings in evaluating ADE, especially in identifying gender differences in the occurrence of ADE. Recent research findings have indicated a significant correlation between disproportionality analyses and risk estimation in clinical studies (Maciá-Martínez et al., 2016; Khouri et al., 2021; Li et al., 2023). Therefore, gender differences in ADE should be explored through post-market safety monitoring of drugs, thereby providing decision-making support for personalized medication guidance and improving the level of rational clinical medication.

### 4.1 Drug abuse and addiction

Ketamine is easily used for entertainment because of its dissociative properties, causing concerns regarding drug abuse and the possibility of addiction. A recent study has found that euphoria, relaxation, and drunkenness are adverse reactions associated with the use of esketamine (Jiang et al., 2023), consistent with the ADE signals identified in this study, and have high ROR values. Similarly, ketamine has become a popular recreational drug owing to its psychotropic effects, including a dream-like state (Van Amsterdam and Van Den Brink, 2022). These psychotropic effects might increase the risk of drug abuse. However, in our study, there were no sex differences in these psychotropic effects. Christian et al. suggested that ketamine reinforces initial self-administration but does not induce synaptic plasticity, which is typically observed with addictive drugs in mice (Simmler et al., 2022). The number of RCTs showing that sub-anesthetic esketamine may not be related to drug abuse (Chiappini et al., 2023; Reif et al., 2023). But some studies have shown that esketamine, (S)-enantiomer of ketamine, has the potential for drug abuse (Nguyen et al., 2023). Esketamine can produce euphoric mood, dissociation, feeling drunk, hallucinations, and other mental states and perceptual changes, which may be abused by some people for entertainment or mental stimulation (Gastaldon et al., 2021). Other reasons for this concern are that some patients have developed drug dependence on ketamine or other substances, as well as recognition of the diversion and misuse of prescription drugs (Kim et al., 2019). The FDA added a REMS to manage possible risks while ensuring its benefits. However, due to the lack of detailed information on drug abuse populations in the FAERS database, which collects ADE information through spontaneous reporting, it is impossible to determine the actual situation of the ADE of concern. In contrast, an increasing number of studies have shown that another (R)-enantiomer, arketamine, has greater efficacy and long-lasting antidepressant-like effects than esketamine (Yang et al., 2015). Importantly, in some animal and human studies, the side effects of arketamine, namely, its psychiatric effects and the risk of abuse, were smaller than those of ketamine and esketamine (Chang et al., 2019; Zhang et al., 2022). Drug signal monitoring, crucial for human health, leverages algorithms to enhance safety detection and uncover risks, yet faces challenges with data quality and false positives. Enhanced by standardization, global collaboration, and regulatory backing, its effectiveness in worldwide drug safety surveillance can be amplified.

### 4.2 Psychiatric and nervous system

This study found a high rate of suicide. Suicide attempts are high-risk signals in men who use esketamine. However, suicidal ideation was a high-risk signal for women, and agitation was a high-risk signal for men with ketamine, consistent with previous research results (Gastaldon et al., 2021). The side effects of antidepressant-dose ketamine, including confusion/agitation, are tolerable and limited to treatment period (Yavi et al., 2022). In the treatment of bipolar depression, the risk of mood switches is an important safety concern. Some literatures suggest that ketamine and esketamine have potential in the treatment of bipolar depression and psychological symptoms have not worsened (Gałuszko-Wȩgielnik et al., 2023; Martinotti et al., 2023). According to systematic reviews and meta-analyses, ketamine and/or esketamine can quickly reduce suicidal ideation (Wang et al., 2021; Chen et al., 2023). However, there is a lack of evidence to prove whether ketamine or esketamine can more persistently reduce suicidal ideation and the decrease in suicide completion rate after 6 weeks (Phillips et al., 2020). Another study has shown that after 24 h and/or 25 days of administration, there was no significant reduction in suicidal ideation compared to placebo (Canuso et al., 2019). A recent study suggested that esketamine could affect brain plasticity and neural networks, thereby influencing mental health during long-term use (Jiang et al., 2023). Several different explanations for the growing trend on overall suicidality and suicidal attempts in patients treated with ketamine and its enantiomers have been described, such as unknown baseline features, comorbidity and its severity, concomitant use of other substances, withdrawal symptom, dissociation, feeling drunk, delayed psychiatric reactions, and ineffectiveness in treating TRD (Schatzberg, 2019; Beck et al., 2020; Gastaldon et al., 2021). However, regardless of the reason, clinicians should be vigilant of such serious complications and conduct research on the long-term effects and risks. Unfortunately, due to too many confounding factors, our research results cannot be strongly compared with clinical trial results. Among the ADEs in esketamine, psychological and neurological symptoms are the most frequent. These datas are consistent with RCT research and real-world data, for example, effects on the psychological and nervous systems are reported more frequently in the elderly population treated with esketamine (d'Andrea et al., 2023). At present, no gender differences in neurological symptoms have been found.

### 4.3 Cardiovascular system

Ketamine can activate the sympathetic nervous system and produce brief cardiovascular stimulation, leading to increased blood pressure and heart rate. The heart is directly inhibited when used at large doses. Usually, after cessation of drug infusion, the cardiovascular inhibitory effect is more pronounced, resulting in a decreased cardiac output (Szarmach et al., 2019). This study detected ADEs related to hypertension and bradycardia, which were all reported as high-risk signals in males, consistent with previous research (Singh et al., 2016; Jones et al., 2022; Nikayin et al., 2022). Therefore, particular attention should be paid to the cardiovascular system conditions of patients receiving ketamine and esketamine, such as heart rate and blood pressure, particularly in hypertensive patients with poor blood pressure control and high-dose drug use.

### 4.4 Hepatobiliary and urinary systems

Ketamine-related abdominal organ damage mainly manifests in the urinary (Chan et al., 2022) and hepatobiliary systems (Cotter et al., 2021). It is primarily metabolized in the liver after entering the human body. Its decomposition products and original compounds are metabolized by the kidneys and eliminated from the body through the urine. In ketamine abuse, drug concentration increases and stimulates the liver, gallbladder, and urinary system for extended periods, leading to abdominal organ damage. AEs associated with the urinary system in males included urinary retention and cystitis, whereas sterile pyuria, increased transaminase levels, and sclerosing cholangitis were identified as high-risk signals in females, consistent with previous studies (Wise et al., 2015). In this study, the high-risk signals for abdominal organ damage were mainly reported with ketamine, which may be related to its susceptibility to abuse and long-term and repeated use (Cotter et al., 2021). Another study suggested that female ketamine users self-reported significantly greater levels of severity of urinary discomfort than did male users (Chen et al., 2014). In the present study, both male and female patients experienced urinary system damage; however, their symptoms differed. This may be related to the hormonal changes and physiological anatomical structures in women. Regular screening, and liver and kidney function follow-up should be conducted in high-risk populations. These results will enable individualized recommendations for ketamine abusers, including different screening and treatment methods for men and women.

### 4.5 Others

The therapeutic product effect decreased, and toxicity to various agents was found to be a high-risk signal for males. Clinicians should be vigilant of male users.

## 5 Conclusion

This study conducted signal mining using the FAERS database and performed an exploratory analysis of the gender differences in ketamine and esketamine ADE signals. These results can assist healthcare professionals in developing personalized treatment plans based on sex-based differences. Adequate measures to reduce the occurrence of ADE are crucial to improve medication safety.

Nevertheless, this study still has certain limitations. Firstly, the reports of this study mostly come from the United States, and cannot accurately reflect the ADE in different population. Signal mining models may overfit training data, resulting in insufficient generalization ability in new data or actual situations. Secondly, when analyzing ADE signals, there may be uncontrollable confounding factors such as age, gender, underlying disease, lifestyle, and combination therapy that affect the accuracy of the results. Thirdly, in the spontaneous reporting system, it is inevitable that there will be duplication, underreporting, omission, and inaccuracy in the report, which may lead to biased research results. Fourthly, social determinants such as socioeconomic status, education level, and availability of medical resources may affect the occurrence and reporting of ADE. Therefore, the gender differences in ADE signals generated by data mining require further evaluation, validation, and subsequent research. This study provides ideas for future signal mining based on patient risk factors.

## Data Availability

The original contributions presented in the study are included in the article/Supplementary Material, further inquiries can be directed to the corresponding authors.

## Abbreviations

ADE, adverse drug event; FAERS, Food and Drug Administration on Adverse Event Reporting System; ROR, reporting odds ratio; CI, confidence interval; FDA, Food and Drug Administration; MedDRA, medical dictionary for regulatory activities; PT, preferred term; SOC, system organ class; PRR, proportional reporting ratio; BCPNN, Bayesian confidence propagation neural network; MGPS, multi-item gamma Poisson shrinker; REMS, Risk Evaluation and Mitigation Strategy; MHRA, medicines and healthcare products regulatory agency; RCT, Randomized Controlled Trial.
