# Supplementary material for: Comparing the adverse effects of ketamine and esketamine between genders using FAERS data
Source: Front Pharmacol. 2024 Jul 12;15:1329436. doi: 10.3389/fphar.2024.1329436 (PMC11272469; doi:10.3389/fphar.2024.1329436)
Supplement: Supplementary file 1 [file Table1.DOCX]

***Supplementary Material***

***Comparing the adverse effects of* ketamine *and esketamine between genders using FAERS Data***

**SUPPLEMENTARY TABLE 1.** Preferred Terms of ADEs related to Esketamine in the ROR values.

| Preferred Terms (PTs) | Cases | ROR | PRR | X^2^ | EBGM05 | IC025 | Unexpected signal |
| --- | --- | --- | --- | --- | --- | --- | --- |
| Dissociation | 921 | 1923.92 | 1779.35 | 788599.35 | 780.03 | 8.68 | / |
| Dissociative disorder | 37 | 354.76 | 353.70 | 10720.23 | 204.34 | 4.57 | / |
| Sedation | 593 | 155.72 | 148.23 | 79613.00 | 124.91 | 6.67 | / |
| Morbid thoughts | 13 | 86.78 | 86.69 | 1046.28 | 47.18 | 2.80 | / |
| Flashback | 4 | 61.84 | 61.82 | 230.73 | 21.97 | 0.91 | Yes |
| Derealisation | 17 | 50.36 | 50.29 | 797.11 | 30.13 | 3.05 | / |
| Euphoric mood | 75 | 47.19 | 46.91 | 3277.41 | 36.26 | 4.51 | / |
| Tachyphylaxis | 3 | 46.82 | 46.80 | 130.78 | 14.45 | 0.44 | Yes |
| Psychogenic seizure | 7 | 45.60 | 45.58 | 297.01 | 20.94 | 1.75 | / |
| Autoscopy | 7 | 42.90 | 42.88 | 279.06 | 19.74 | 1.74 | / |
| Drug monitoring procedure incorrectly performed | 7 | 41.66 | 41.64 | 270.84 | 19.19 | 1.74 | / |
| Suicidal ideation | 536 | 41.58 | 39.80 | 19819.98 | 35.63 | 5.05 | / |
| Illusion | 11 | 40.64 | 40.61 | 414.76 | 21.80 | 2.39 | / |
| Major depression | 50 | 39.63 | 39.47 | 1831.23 | 29.12 | 4.06 | / |
| Depressive symptom | 21 | 33.07 | 33.01 | 639.17 | 21.02 | 3.12 | / |
| Feeling drunk | 33 | 32.69 | 32.60 | 991.39 | 22.66 | 3.56 | / |
| Feeling of relaxation | 3 | 32.22 | 32.22 | 89.01 | 10.09 | 0.41 | / |
| Negative thoughts | 11 | 25.72 | 25.69 | 257.08 | 13.95 | 2.22 | / |
| Nasal discomfort | 31 | 24.95 | 24.89 | 700.27 | 17.20 | 3.31 | / |
| Flat affect | 6 | 24.45 | 24.44 | 132.92 | 10.76 | 1.39 | / |
| Dysphoria | 11 | 22.26 | 22.24 | 220.17 | 12.11 | 2.16 | / |
| Conversion disorder | 5 | 21.88 | 21.88 | 98.31 | 8.94 | 1.10 | Yes |
| Alcohol poisoning | 7 | 20.00 | 19.99 | 124.80 | 9.38 | 1.53 | / |
| Self-injurious ideation | 12 | 18.92 | 18.90 | 201.15 | 10.58 | 2.18 | / |
| Suicide attempt | 171 | 17.45 | 17.22 | 2588.17 | 14.65 | 3.74 | / |
| Panic attack | 96 | 16.70 | 16.57 | 1391.62 | 13.42 | 3.53 | / |
| Essential tremor | 3 | 16.49 | 16.48 | 43.20 | 5.24 | 0.30 | Yes |
| Panic disorder | 10 | 15.58 | 15.57 | 135.10 | 8.28 | 1.86 | / |
| Feeling of despair | 12 | 15.05 | 15.03 | 155.80 | 8.44 | 2.04 | / |
| Hyperacusis | 9 | 14.49 | 14.48 | 111.95 | 7.45 | 1.70 | / |
| Agoraphobia | 3 | 14.34 | 14.34 | 36.91 | 4.56 | 0.27 | Yes |
| Bipolar I disorder | 4 | 14.20 | 14.20 | 48.65 | 5.26 | 0.66 | / |
| Logorrhoea | 6 | 13.94 | 13.94 | 71.45 | 6.19 | 1.19 | / |
| Inappropriate affect | 3 | 13.34 | 13.34 | 33.96 | 4.25 | 0.25 | / |
| Depression suicidal | 9 | 13.02 | 13.01 | 99.01 | 6.70 | 1.64 | / |
| Disturbance in social behaviour | 6 | 12.81 | 12.80 | 64.79 | 5.69 | 1.15 | / |
| Fear of death | 4 | 12.53 | 12.53 | 42.12 | 4.65 | 0.62 | / |
| Device dispensing error | 4 | 12.30 | 12.30 | 41.20 | 4.57 | 0.62 | / |
| Motion sickness | 4 | 11.99 | 11.98 | 39.98 | 4.45 | 0.61 | / |
| Post-traumatic stress disorder | 18 | 11.57 | 11.55 | 172.30 | 7.22 | 2.22 | / |
| Cystitis interstitial | 3 | 11.41 | 11.41 | 28.28 | 3.64 | 0.21 | / |
| Hypomania | 5 | 11.35 | 11.34 | 46.83 | 4.68 | 0.87 | / |
| Disinhibition | 3 | 11.28 | 11.28 | 27.90 | 3.60 | 0.21 | / |
| Depression | 380 | 10.87 | 10.57 | 3279.63 | 9.48 | 3.21 | / |
| Bladder spasm | 3 | 10.86 | 10.86 | 26.67 | 3.47 | 0.20 | / |
| Staring | 3 | 10.32 | 10.31 | 25.08 | 3.30 | 0.18 | / |
| Somatic symptom disorder | 4 | 10.18 | 10.18 | 32.90 | 3.79 | 0.55 | / |
| Sensory disturbance | 23 | 10.16 | 10.14 | 188.44 | 6.69 | 2.28 | / |
| Therapeutic product effect increased | 5 | 9.62 | 9.61 | 38.37 | 3.97 | 0.80 | / |
| Hypoaesthesia oral | 23 | 9.53 | 9.52 | 174.32 | 6.28 | 2.22 | / |
| Crying | 48 | 9.47 | 9.44 | 360.09 | 7.06 | 2.59 | / |
| Mania | 21 | 9.46 | 9.44 | 157.64 | 6.12 | 2.15 | / |
| Hallucination, visual | 37 | 9.43 | 9.40 | 276.33 | 6.77 | 2.47 | / |
| Catatonia | 8 | 8.86 | 8.86 | 55.45 | 4.40 | 1.27 | / |
| Fear | 28 | 8.70 | 8.68 | 189.44 | 5.96 | 2.24 | / |
| Hangover | 5 | 8.57 | 8.57 | 33.26 | 3.54 | 0.74 | / |
| Hallucination, auditory | 21 | 8.17 | 8.16 | 131.28 | 5.29 | 2.00 | / |
| Drug monitoring procedure not performed | 5 | 8.02 | 8.01 | 30.54 | 3.31 | 0.70 | / |
| Sedation complication | 6 | 7.95 | 7.94 | 36.25 | 3.55 | 0.90 | / |
| Bipolar disorder | 13 | 7.90 | 7.90 | 77.92 | 4.56 | 1.63 | / |
| Hyperventilation | 7 | 7.80 | 7.80 | 41.28 | 3.69 | 1.05 | / |
| Completed suicide | 97 | 7.59 | 7.53 | 547.70 | 6.14 | 2.52 | / |
| Respiratory rate decreased | 4 | 7.50 | 7.50 | 22.43 | 2.80 | 0.41 | / |
| Hallucination | 99 | 7.38 | 7.33 | 539.67 | 5.99 | 2.49 | / |
| Screaming | 6 | 7.35 | 7.35 | 32.77 | 3.28 | 0.85 | / |
| Bladder pain | 4 | 7.29 | 7.29 | 21.60 | 2.72 | 0.39 | / |
| Substance abuse | 13 | 6.99 | 6.98 | 66.39 | 4.04 | 1.51 | / |
| Psychotic symptom | 4 | 6.98 | 6.98 | 20.40 | 2.60 | 0.37 | / |
| Electric shock sensation | 5 | 6.93 | 6.93 | 25.27 | 2.87 | 0.62 | / |
| Blood pressure increased | 211 | 6.89 | 6.79 | 1039.23 | 5.90 | 2.52 | / |
| Tearfulness | 4 | 6.88 | 6.88 | 20.00 | 2.57 | 0.36 | / |
| Affect lability | 8 | 6.75 | 6.74 | 38.98 | 3.36 | 1.07 | / |
| Nonspecific reaction | 6 | 6.49 | 6.49 | 27.73 | 2.90 | 0.77 | / |
| Alcohol abuse | 3 | 6.31 | 6.30 | 13.34 | 2.02 | -0.01 | / |
| Taste disorder | 44 | 6.27 | 6.25 | 193.43 | 4.63 | 2.05 | / |
| Paranoia | 15 | 6.23 | 6.22 | 65.51 | 3.73 | 1.50 | / |
| Hypertension | 237 | 6.21 | 6.11 | 1011.82 | 5.35 | 2.39 | / |
| Micturition urgency | 12 | 5.88 | 5.88 | 48.44 | 3.33 | 1.29 | / |
| Hypopnoea | 4 | 5.87 | 5.87 | 16.10 | 2.19 | 0.28 | / |
| Loss of therapeutic response | 4 | 5.78 | 5.78 | 15.75 | 2.16 | 0.27 | / |
| Unresponsive to stimuli | 23 | 5.76 | 5.75 | 89.95 | 3.81 | 1.67 | / |
| Panic reaction | 7 | 5.75 | 5.75 | 27.36 | 2.73 | 0.83 | / |
| Intentional self-injury | 32 | 5.74 | 5.73 | 124.58 | 4.04 | 1.82 | / |
| Anxiety | 306 | 5.74 | 5.62 | 1163.86 | 5.00 | 2.30 | / |
| Psychotic disorder | 24 | 5.68 | 5.67 | 92.05 | 3.79 | 1.67 | / |
| Akathisia | 12 | 5.62 | 5.62 | 45.43 | 3.18 | 1.25 | / |
| Underdose | 78 | 5.50 | 5.47 | 284.49 | 4.37 | 2.04 | / |
| Alcoholism | 6 | 5.44 | 5.44 | 21.66 | 2.43 | 0.64 | / |
| Blood pressure diastolic increased | 7 | 5.32 | 5.32 | 24.45 | 2.52 | 0.76 | / |
| Hallucinations, mixed | 4 | 5.27 | 5.27 | 13.78 | 1.97 | 0.21 | / |
| Dysgeusia | 56 | 4.90 | 4.88 | 172.50 | 3.74 | 1.81 | / |
| Thinking abnormal | 14 | 4.87 | 4.86 | 42.83 | 2.87 | 1.20 | / |
| Anger | 24 | 4.83 | 4.82 | 72.57 | 3.22 | 1.48 | / |
| Concussion | 7 | 4.65 | 4.65 | 20.00 | 2.21 | 0.65 | / |
| Poisoning | 7 | 4.47 | 4.47 | 18.81 | 2.12 | 0.62 | / |
| Dependence | 9 | 4.47 | 4.47 | 24.17 | 2.32 | 0.81 | / |
| Emotional disorder | 22 | 4.35 | 4.35 | 56.59 | 2.85 | 1.32 | / |
| Vomiting | 338 | 4.30 | 4.21 | 830.97 | 3.77 | 1.90 | / |
| Feeling abnormal | 187 | 4.28 | 4.23 | 460.89 | 3.65 | 1.84 | / |
| Agitation | 43 | 4.16 | 4.15 | 102.53 | 3.07 | 1.51 | / |
| Near death experience | 5 | 4.08 | 4.08 | 11.61 | 1.69 | 0.25 | / |
| Diplopia | 17 | 4.07 | 4.07 | 39.22 | 2.52 | 1.11 | / |
| Psychiatric symptom | 5 | 4.04 | 4.04 | 11.43 | 1.68 | 0.24 | / |
| Depressed mood | 40 | 4.00 | 3.99 | 89.34 | 2.92 | 1.44 | / |
| Hypokinesia | 12 | 3.93 | 3.93 | 26.12 | 2.22 | 0.88 | / |
| Frustration tolerance decreased | 9 | 3.86 | 3.86 | 19.01 | 2.00 | 0.67 | / |
| Mood altered | 16 | 3.84 | 3.84 | 33.55 | 2.35 | 1.01 | / |
| Adverse event | 56 | 3.75 | 3.74 | 112.30 | 2.87 | 1.45 | / |
| Paralysis | 9 | 3.67 | 3.67 | 17.41 | 1.90 | 0.62 | / |
| Sensory loss | 5 | 3.63 | 3.63 | 9.51 | 1.51 | 0.15 | / |
| Abnormal behaviour | 16 | 3.63 | 3.62 | 30.34 | 2.21 | 0.95 | / |
| Nightmare | 18 | 3.56 | 3.56 | 33.01 | 2.24 | 0.98 | / |
| Treatment noncompliance | 32 | 3.44 | 3.44 | 55.17 | 2.42 | 1.17 | / |
| Dysarthria | 20 | 3.40 | 3.40 | 33.77 | 2.19 | 0.98 | / |
| Affective disorder | 5 | 3.37 | 3.37 | 8.33 | 1.40 | 0.09 | / |
| Eating disorder | 14 | 3.32 | 3.32 | 22.61 | 1.96 | 0.77 | / |
| Withdrawal syndrome | 28 | 3.32 | 3.31 | 45.15 | 2.28 | 1.08 | / |
| Disorientation | 19 | 3.26 | 3.26 | 29.66 | 2.07 | 0.90 | / |
| Dizziness | 270 | 3.18 | 3.13 | 394.45 | 2.77 | 1.46 | / |
| Pollakiuria | 23 | 3.16 | 3.15 | 33.81 | 2.09 | 0.94 | / |
| Therapeutic response decreased | 32 | 3.13 | 3.12 | 46.06 | 2.20 | 1.05 | / |
| Mental impairment | 13 | 3.12 | 3.12 | 18.70 | 1.81 | 0.66 | / |
| Vertigo | 32 | 3.11 | 3.10 | 45.49 | 2.19 | 1.04 | / |
| Therapeutic product effect decreased | 58 | 3.02 | 3.01 | 77.91 | 2.32 | 1.16 | / |
| Mental disorder | 26 | 2.97 | 2.97 | 33.84 | 2.02 | 0.91 | / |
| Muscle twitching | 11 | 2.92 | 2.92 | 13.85 | 1.61 | 0.50 | / |
| Irritability | 23 | 2.87 | 2.86 | 27.84 | 1.90 | 0.82 | / |
| Nausea | 392 | 2.86 | 2.80 | 456.88 | 2.53 | 1.33 | / |
| Hypersomnia | 15 | 2.82 | 2.82 | 17.55 | 1.69 | 0.61 | / |
| Psychomotor hyperactivity | 7 | 2.80 | 2.80 | 8.09 | 1.33 | 0.17 | / |
| Aggression | 18 | 2.71 | 2.71 | 19.41 | 1.70 | 0.65 | / |
| Urinary incontinence | 13 | 2.70 | 2.70 | 13.90 | 1.57 | 0.49 | / |
| Dystonia | 6 | 2.68 | 2.68 | 6.32 | 1.20 | 0.02 | / |
| Medication error | 17 | 2.62 | 2.62 | 17.04 | 1.63 | 0.58 | / |
| Depressed level of consciousness | 19 | 2.58 | 2.58 | 18.32 | 1.64 | 0.61 | / |
| Transient ischaemic attack | 11 | 2.46 | 2.46 | 9.54 | 1.36 | 0.30 | / |
| Product dose omission issue | 290 | 2.45 | 2.41 | 241.67 | 2.14 | 1.09 | / |
| Product administration error | 18 | 2.43 | 2.43 | 15.14 | 1.53 | 0.51 | / |
| Abnormal dreams | 7 | 2.41 | 2.41 | 5.75 | 1.15 | 0.01 | / |
| Hypoaesthesia | 62 | 2.39 | 2.38 | 49.89 | 1.86 | 0.86 | / |
| Somnolence | 84 | 2.34 | 2.33 | 63.67 | 1.88 | 0.88 | / |
| Hyperhidrosis | 48 | 2.27 | 2.26 | 33.86 | 1.70 | 0.73 | / |
| Urinary retention | 13 | 2.22 | 2.22 | 8.74 | 1.29 | 0.26 | / |
| Speech disorder | 20 | 2.21 | 2.21 | 13.25 | 1.42 | 0.43 | / |
| Lethargy | 19 | 2.12 | 2.12 | 11.26 | 1.35 | 0.36 | / |
| Blood pressure decreased | 25 | 2.11 | 2.10 | 14.49 | 1.42 | 0.45 | / |
| Loss of consciousness | 43 | 2.08 | 2.08 | 24.00 | 1.54 | 0.58 | / |
| Road traffic accident | 14 | 2.05 | 2.04 | 7.46 | 1.21 | 0.19 | / |
| Nervousness | 16 | 2.03 | 2.03 | 8.30 | 1.24 | 0.23 | / |
| Feeling cold | 10 | 2.02 | 2.02 | 5.12 | 1.08 | 0.01 | / |
| Throat irritation | 16 | 2.01 | 2.01 | 8.07 | 1.23 | 0.22 | / |

**SUPPLEMENTARY TABLE 1.** Preferred Terms of ADEs related to Ketamine in the ROR values.

| Preferred Terms (PTs) | Cases | ROR | PRR | X^2^ | EBGM05 | IC025 | Unexpected signal |
| --- | --- | --- | --- | --- | --- | --- | --- |
| Ureteral polyp | 18 | 19136.17 | 19077.04 | 105646.43 | 2551.58 | 3.38 | / |
| Cystitis ulcerative | 34 | 13807.98 | 13727.39 | 178180.76 | 3040.75 | 4.50 | / |
| Biopsy bladder abnormal | 11 | 11680.25 | 11658.19 | 53986.59 | 1973.94 | 2.53 | / |
| Reduced bladder capacity | 31 | 6445.01 | 6410.71 | 113131.74 | 2288.25 | 4.37 | / |
| Sterile pyuria | 15 | 3863.89 | 3853.95 | 39723.25 | 1438.55 | 3.16 | / |
| Contracted bladder | 36 | 2625.04 | 2608.83 | 71764.62 | 1372.05 | 4.65 | / |
| Lower urinary tract symptoms | 42 | 1749.70 | 1737.10 | 60482.83 | 1033.53 | 4.91 | / |
| Ureteritis | 14 | 1166.54 | 1163.74 | 14301.48 | 585.04 | 3.09 | / |
| Bladder necrosis | 5 | 800.56 | 799.88 | 3645.48 | 292.10 | 1.34 | / |
| Bladder hypertrophy | 21 | 627.00 | 624.75 | 12180.27 | 373.32 | 3.77 | / |
| Biliary dilatation | 61 | 450.96 | 446.25 | 25744.85 | 327.32 | 5.38 | / |
| Biliary tract disorder | 54 | 420.49 | 416.61 | 21340.74 | 301.83 | 5.20 | / |
| Pyonephrosis | 3 | 397.64 | 397.44 | 1133.21 | 119.25 | 0.50 | / |
| Bladder neck obstruction | 4 | 317.18 | 316.96 | 1214.46 | 112.59 | 0.98 | / |
| Ureteral disorder | 13 | 315.62 | 314.92 | 3922.38 | 174.47 | 2.96 | / |
| Ureteric stenosis | 23 | 305.43 | 304.23 | 6710.61 | 193.62 | 3.88 | / |
| Urogenital fistula | 5 | 305.25 | 304.99 | 1462.36 | 120.60 | 1.36 | / |
| Dissociative disorder | 14 | 241.84 | 241.26 | 3257.10 | 137.84 | 3.07 | / |
| Ureteric injury | 3 | 240.09 | 239.96 | 694.25 | 74.06 | 0.51 | / |
| Hydronephrosis | 153 | 222.31 | 216.50 | 32005.32 | 179.45 | 6.24 | / |
| Waxy flexibility | 4 | 216.17 | 216.02 | 834.82 | 78.07 | 0.98 | / |
| Intra-abdominal pressure increased | 3 | 215.67 | 215.56 | 624.81 | 66.82 | 0.51 | / |
| Hydroureter | 7 | 211.47 | 211.21 | 1428.93 | 97.32 | 1.92 | / |
| Urge incontinence | 16 | 192.41 | 191.88 | 2970.97 | 114.25 | 3.26 | / |
| Urinary tract disorder | 74 | 183.82 | 181.49 | 13005.47 | 140.95 | 5.39 | / |
| Airway complication of anaesthesia | 4 | 172.27 | 172.16 | 667.10 | 62.69 | 0.98 | / |
| Cystitis interstitial | 26 | 167.49 | 166.75 | 4201.19 | 110.85 | 3.98 | / |
| Biliary cyst | 3 | 153.31 | 153.23 | 445.65 | 48.04 | 0.51 | / |
| Ureteric obstruction | 19 | 141.27 | 140.82 | 2594.63 | 87.97 | 3.48 | / |
| Renal papillary necrosis | 3 | 136.09 | 136.02 | 395.74 | 42.78 | 0.51 | / |
| Pyuria | 13 | 133.09 | 132.80 | 1674.29 | 75.56 | 2.89 | / |
| Diabetes insipidus | 28 | 126.75 | 126.14 | 3425.43 | 85.52 | 4.02 | / |
| Abdominal compartment syndrome | 7 | 121.52 | 121.37 | 823.87 | 56.73 | 1.89 | / |
| Metaplasia | 6 | 116.53 | 116.41 | 677.23 | 51.29 | 1.63 | / |
| Cystitis noninfective | 14 | 115.86 | 115.58 | 1568.90 | 67.26 | 2.99 | / |
| Suprapubic pain | 5 | 101.02 | 100.94 | 488.92 | 41.29 | 1.33 | / |
| Accidental death | 18 | 95.32 | 95.03 | 1656.20 | 59.02 | 3.33 | / |
| Intensive care unit acquired weakness | 4 | 94.01 | 93.95 | 363.80 | 34.68 | 0.96 | / |
| Hepatobiliary disease | 5 | 93.25 | 93.17 | 450.96 | 38.17 | 1.32 | / |
| Laryngospasm | 25 | 92.79 | 92.40 | 2236.02 | 61.59 | 3.78 | / |
| Delayed recovery from anaesthesia | 8 | 91.79 | 91.66 | 709.72 | 45.16 | 2.08 | / |
| Biliary sepsis | 4 | 90.02 | 89.96 | 348.19 | 33.23 | 0.96 | / |
| Dose calculation error | 4 | 84.84 | 84.79 | 327.92 | 31.35 | 0.96 | / |
| Hypertonic bladder | 21 | 80.93 | 80.64 | 1636.21 | 51.94 | 3.50 | / |
| Cholangitis sclerosing | 14 | 80.23 | 80.04 | 1082.57 | 46.82 | 2.92 | / |
| Vesicoureteric reflux | 3 | 71.09 | 71.05 | 205.47 | 22.61 | 0.49 | / |
| Pneumatosis | 3 | 70.89 | 70.85 | 204.89 | 22.55 | 0.49 | / |
| Necrosis ischaemic | 3 | 70.69 | 70.66 | 204.31 | 22.49 | 0.49 | / |
| Propofol infusion syndrome | 4 | 61.15 | 61.11 | 234.81 | 22.69 | 0.93 | / |
| Maternal exposure during delivery | 4 | 61.04 | 61.00 | 234.37 | 22.64 | 0.93 | / |
| Intrusive thoughts | 4 | 57.13 | 57.10 | 218.98 | 21.21 | 0.93 | / |
| Bladder disorder | 54 | 55.35 | 54.85 | 2836.77 | 41.65 | 4.40 | / |
| Product selection error | 5 | 51.81 | 51.76 | 247.40 | 21.35 | 1.27 | / |
| Bladder dysfunction | 5 | 51.49 | 51.45 | 245.85 | 21.22 | 1.27 | / |
| Cholestatic liver injury | 8 | 49.80 | 49.73 | 379.77 | 24.66 | 1.99 | / |
| Drug abuse | 371 | 48.76 | 45.72 | 16162.42 | 40.93 | 5.19 | / |
| Protrusion tongue | 3 | 48.75 | 48.73 | 139.45 | 15.57 | 0.46 | / |
| Hyperchloraemia | 3 | 45.44 | 45.42 | 129.64 | 14.52 | 0.46 | / |
| Pressure of speech | 3 | 44.34 | 44.31 | 126.35 | 14.17 | 0.46 | / |
| Myocardial fibrosis | 3 | 37.65 | 37.63 | 106.49 | 12.05 | 0.44 | / |
| Hepatic haemorrhage | 3 | 37.15 | 37.13 | 105.02 | 11.89 | 0.44 | / |
| Substance dependence | 3 | 35.44 | 35.43 | 99.95 | 11.35 | 0.43 | / |
| Apnoea | 27 | 34.18 | 34.03 | 862.28 | 23.21 | 3.41 | / |
| Drug screen positive | 26 | 33.11 | 32.97 | 802.90 | 22.33 | 3.36 | / |
| Pulseless electrical activity | 14 | 29.13 | 29.06 | 378.03 | 17.13 | 2.59 | / |
| Dysphoria | 15 | 29.02 | 28.95 | 403.44 | 17.37 | 2.67 | / |
| Cystitis | 87 | 27.75 | 27.35 | 2202.61 | 22.05 | 4.08 | / |
| Bile duct stenosis | 3 | 26.40 | 26.39 | 73.05 | 8.47 | 0.40 | / |
| Micturition urgency | 27 | 25.31 | 25.20 | 625.68 | 17.21 | 3.21 | / |
| Increased bronchial secretion | 5 | 24.94 | 24.92 | 114.48 | 10.33 | 1.14 | / |
| Tonic clonic movements | 3 | 24.78 | 24.77 | 68.23 | 7.95 | 0.39 | / |
| Blood immunoglobulin E increased | 5 | 22.95 | 22.93 | 104.58 | 9.50 | 1.12 | / |
| Heat stroke | 3 | 21.53 | 21.52 | 58.55 | 6.91 | 0.37 | / |
| Renal infarct | 3 | 21.44 | 21.43 | 58.28 | 6.88 | 0.36 | / |
| Hyperthermia malignant | 3 | 21.22 | 21.21 | 57.64 | 6.81 | 0.36 | / |
| Pyelonephritis acute | 4 | 21.22 | 21.21 | 76.84 | 7.93 | 0.78 | / |
| Drug use disorder | 10 | 21.15 | 21.11 | 191.14 | 11.32 | 2.03 | / |
| Reversible cerebral vasoconstriction syndrome | 3 | 20.93 | 20.92 | 56.76 | 6.72 | 0.36 | / |
| Derealisation | 4 | 20.73 | 20.72 | 74.88 | 7.75 | 0.77 | / |
| Respiratory depression | 24 | 19.95 | 19.87 | 429.12 | 13.27 | 2.92 | / |
| Pneumomediastinum | 3 | 19.87 | 19.86 | 53.60 | 6.38 | 0.35 | / |
| Mania | 30 | 18.59 | 18.50 | 495.65 | 12.89 | 3.04 | / |
| Depersonalisation/derealisation disorder | 4 | 18.00 | 17.99 | 64.06 | 6.73 | 0.74 | / |
| Kidney fibrosis | 3 | 17.18 | 17.17 | 45.61 | 5.52 | 0.32 | / |
| Renal function test abnormal | 7 | 17.05 | 17.03 | 105.42 | 8.09 | 1.48 | / |
| Sedation complication | 3 | 16.24 | 16.23 | 42.80 | 5.22 | 0.31 | / |
| Hyperaemia | 3 | 16.20 | 16.19 | 42.68 | 5.21 | 0.31 | / |
| Haematuria | 55 | 15.99 | 15.85 | 764.17 | 12.13 | 3.26 | / |
| Anaesthetic complication | 4 | 15.27 | 15.26 | 53.22 | 5.71 | 0.69 | / |
| Biliary colic | 5 | 15.03 | 15.02 | 65.33 | 6.24 | 0.99 | / |
| Nystagmus | 8 | 14.81 | 14.79 | 102.70 | 7.38 | 1.58 | / |
| Hypernatraemia | 7 | 14.78 | 14.76 | 89.67 | 7.02 | 1.42 | / |
| Urinary tract obstruction | 4 | 14.52 | 14.51 | 50.24 | 5.43 | 0.68 | / |
| Incorrect drug administration rate | 5 | 14.17 | 14.15 | 61.03 | 5.88 | 0.97 | / |
| Trismus | 7 | 14.12 | 14.10 | 85.07 | 6.70 | 1.40 | / |
| Stress cardiomyopathy | 7 | 14.10 | 14.08 | 84.95 | 6.70 | 1.40 | / |
| Pollakiuria | 55 | 13.86 | 13.73 | 648.75 | 10.51 | 3.09 | / |
| Salivary hypersecretion | 13 | 13.84 | 13.81 | 154.25 | 8.00 | 2.08 | / |
| Corneal oedema | 3 | 13.68 | 13.68 | 35.19 | 4.40 | 0.27 | / |
| Biliary obstruction | 4 | 13.67 | 13.66 | 46.85 | 5.11 | 0.66 | / |
| Liver abscess | 4 | 13.56 | 13.56 | 46.44 | 5.07 | 0.66 | / |
| Major depression | 9 | 13.14 | 13.12 | 100.63 | 6.81 | 1.65 | / |
| Cholangitis | 7 | 13.14 | 13.12 | 78.27 | 6.24 | 1.36 | / |
| Dissociation | 6 | 13.08 | 13.07 | 66.79 | 5.86 | 1.17 | / |
| Dysuria | 47 | 13.03 | 12.94 | 517.15 | 9.69 | 2.95 | / |
| Pulse absent | 6 | 12.26 | 12.25 | 61.88 | 5.49 | 1.14 | / |
| Product label confusion | 6 | 12.00 | 11.99 | 60.36 | 5.37 | 1.13 | / |
| Hypoxia | 37 | 11.40 | 11.34 | 348.51 | 8.19 | 2.68 | / |
| Hypomania | 4 | 10.92 | 10.92 | 35.98 | 4.09 | 0.58 | / |
| Cystitis haemorrhagic | 4 | 10.74 | 10.74 | 35.27 | 4.02 | 0.57 | / |
| Liver function test abnormal | 32 | 10.45 | 10.39 | 271.48 | 7.33 | 2.51 | / |
| Drug tolerance | 5 | 10.40 | 10.39 | 42.37 | 4.31 | 0.84 | / |
| Bladder pain | 3 | 10.13 | 10.13 | 24.65 | 3.26 | 0.18 | / |
| Nocturia | 11 | 10.05 | 10.03 | 89.37 | 5.55 | 1.68 | / |
| Substance abuse | 9 | 9.97 | 9.96 | 72.48 | 5.17 | 1.48 | / |
| Brain death | 3 | 9.93 | 9.93 | 24.06 | 3.20 | 0.17 | / |
| Muscle rigidity | 11 | 9.63 | 9.61 | 84.77 | 5.31 | 1.65 | / |
| Respiratory rate decreased | 3 | 9.50 | 9.50 | 22.79 | 3.06 | 0.16 | / |
| Hallucination, visual | 18 | 9.44 | 9.41 | 135.22 | 5.92 | 2.04 | / |
| Coma scale abnormal | 3 | 9.20 | 9.20 | 21.90 | 2.96 | 0.15 | / |
| Cardiac arrest | 75 | 9.19 | 9.08 | 539.79 | 7.23 | 2.70 | / |
| Paradoxical drug reaction | 3 | 9.08 | 9.07 | 21.52 | 2.92 | 0.14 | / |
| Brain oedema | 11 | 9.06 | 9.05 | 78.65 | 5.00 | 1.60 | / |
| Respiratory arrest | 26 | 8.84 | 8.81 | 179.87 | 5.99 | 2.21 | / |
| Hypoventilation | 3 | 8.78 | 8.78 | 20.66 | 2.83 | 0.13 | / |
| Partial seizures | 4 | 8.35 | 8.35 | 25.84 | 3.13 | 0.46 | / |
| Respiratory acidosis | 3 | 8.34 | 8.34 | 19.35 | 2.68 | 0.11 | / |
| Ventricular fibrillation | 9 | 8.15 | 8.14 | 56.28 | 4.23 | 1.33 | / |
| Unresponsive to stimuli | 20 | 8.00 | 7.97 | 121.91 | 5.13 | 1.95 | / |
| Aspiration | 8 | 7.99 | 7.98 | 48.83 | 3.99 | 1.20 | / |
| Systemic inflammatory response syndrome | 3 | 7.93 | 7.93 | 18.15 | 2.55 | 0.09 | / |
| Hypoxic-ischaemic encephalopathy | 3 | 7.78 | 7.78 | 17.71 | 2.51 | 0.08 | / |
| Bradycardia | 41 | 7.78 | 7.73 | 240.36 | 5.68 | 2.29 | / |
| Haemodynamic instability | 5 | 7.47 | 7.46 | 27.95 | 3.10 | 0.67 | / |
| Asphyxia | 6 | 7.35 | 7.34 | 32.86 | 3.29 | 0.85 | / |
| Supraventricular tachycardia | 7 | 7.33 | 7.33 | 38.21 | 3.49 | 1.01 | / |
| Euphoric mood | 8 | 7.30 | 7.29 | 43.42 | 3.64 | 1.14 | / |
| Hallucination, auditory | 11 | 7.25 | 7.24 | 59.14 | 4.00 | 1.42 | / |
| Metabolic acidosis | 21 | 7.20 | 7.18 | 111.66 | 4.67 | 1.87 | / |
| Suicidal behaviour | 3 | 7.05 | 7.05 | 15.57 | 2.27 | 0.04 | / |
| Sedation | 16 | 7.02 | 7.01 | 82.34 | 4.29 | 1.67 | / |
| Parosmia | 5 | 6.96 | 6.95 | 25.46 | 2.89 | 0.62 | / |
| Left ventricular hypertrophy | 3 | 6.95 | 6.94 | 15.25 | 2.24 | 0.04 | / |
| Hallucination | 47 | 6.91 | 6.86 | 235.40 | 5.14 | 2.19 |  |
| Tearfulness | 3 | 6.89 | 6.89 | 15.09 | 2.22 | 0.03 |  |
| Urosepsis | 6 | 6.84 | 6.84 | 29.89 | 3.07 | 0.81 | / |
| Cholestasis | 12 | 6.76 | 6.75 | 58.71 | 3.83 | 1.42 | / |
| Klebsiella infection | 3 | 6.68 | 6.68 | 14.48 | 2.15 | 0.02 | / |
| Blood alkaline phosphatase increased | 17 | 6.63 | 6.61 | 80.90 | 4.10 | 1.65 | / |
| Post-traumatic stress disorder | 4 | 6.58 | 6.58 | 18.92 | 2.47 | 0.34 | / |
| Transaminases increased | 14 | 6.48 | 6.47 | 64.71 | 3.83 | 1.50 | / |
| Screaming | 4 | 6.45 | 6.45 | 18.40 | 2.42 | 0.33 | / |
| Liver injury | 10 | 5.61 | 5.60 | 37.80 | 3.01 | 1.11 | / |
| Delusion | 8 | 5.54 | 5.53 | 29.71 | 2.76 | 0.92 | / |
| Poisoning | 7 | 5.52 | 5.52 | 25.89 | 2.63 | 0.80 | / |
| Medication error | 29 | 5.42 | 5.40 | 103.98 | 3.75 | 1.71 | / |
| Posterior reversible encephalopathy syndrome | 5 | 5.36 | 5.35 | 17.69 | 2.23 | 0.45 | / |
| Delirium | 17 | 5.34 | 5.33 | 59.73 | 3.31 | 1.42 | / |
| Acidosis | 4 | 5.30 | 5.30 | 13.95 | 1.99 | 0.22 | / |
| Inappropriate antidiuretic hormone secretion | 5 | 5.29 | 5.29 | 17.38 | 2.20 | 0.45 | / |
| Circulatory collapse | 9 | 5.18 | 5.18 | 30.32 | 2.69 | 0.95 | / |
| Generalised tonic-clonic seizure | 13 | 5.15 | 5.14 | 43.38 | 2.98 | 1.22 | / |
| Wrong product administered | 8 | 5.11 | 5.10 | 26.39 | 2.55 | 0.85 |  |
| Ventricular tachycardia | 8 | 4.95 | 4.94 | 25.15 | 2.47 | 0.82 |  |
| Hyperaesthesia | 4 | 4.74 | 4.74 | 11.79 | 1.78 | 0.15 | / |
| Mental status changes | 13 | 4.74 | 4.73 | 38.22 | 2.74 | 1.13 |  |
| Accidental exposure to product by child | 4 | 4.73 | 4.73 | 11.75 | 1.77 | 0.15 |  |
| Status epilepticus | 5 | 4.67 | 4.67 | 14.41 | 1.94 | 0.36 |  |
| Paranoia | 8 | 4.65 | 4.65 | 22.91 | 2.32 | 0.76 |  |
| Psychotic disorder | 13 | 4.51 | 4.50 | 35.44 | 2.61 | 1.08 |  |
| Depressed level of consciousness | 17 | 4.40 | 4.39 | 44.58 | 2.73 | 1.20 |  |
| Ileus | 5 | 4.38 | 4.37 | 13.00 | 1.82 | 0.31 |  |
| Agitation | 32 | 4.30 | 4.28 | 80.60 | 3.02 | 1.46 |  |
| Sinus tachycardia | 6 | 4.29 | 4.29 | 15.12 | 1.92 | 0.45 |  |
| Alanine aminotransferase increased | 26 | 4.29 | 4.27 | 65.21 | 2.91 | 1.37 |  |
| Bronchospasm | 6 | 4.24 | 4.23 | 14.82 | 1.90 | 0.44 |  |
| Nephropathy | 4 | 4.21 | 4.20 | 9.77 | 1.58 | 0.07 |  |
| Maternal drugs affecting foetus | 4 | 4.20 | 4.20 | 9.74 | 1.57 | 0.06 |  |
| Affect lability | 4 | 4.04 | 4.04 | 9.15 | 1.52 | 0.04 |  |
| Tachypnoea | 5 | 4.00 | 4.00 | 11.25 | 1.66 | 0.24 |  |
| Accidental overdose | 13 | 3.91 | 3.91 | 28.10 | 2.27 | 0.92 |  |
| Hepatic function abnormal | 13 | 3.88 | 3.87 | 27.72 | 2.25 | 0.91 |  |
| Toxicity to various agents | 65 | 3.77 | 3.74 | 130.91 | 2.93 | 1.49 |  |
| Pulmonary oedema | 16 | 3.66 | 3.65 | 30.77 | 2.23 | 0.96 |  |
| Oxygen saturation decreased | 18 | 3.65 | 3.64 | 34.53 | 2.29 | 1.01 |  |
| Drug effective for unapproved indication | 6 | 3.60 | 3.60 | 11.27 | 1.62 | 0.30 |  |
| Hepatic cirrhosis | 6 | 3.56 | 3.56 | 11.03 | 1.60 | 0.29 |  |
| Road traffic accident | 14 | 3.50 | 3.50 | 24.95 | 2.07 | 0.84 |  |
| Paralysis | 5 | 3.48 | 3.47 | 8.81 | 1.45 | 0.12 |  |
| Hepatotoxicity | 7 | 3.46 | 3.46 | 12.24 | 1.65 | 0.38 |  |
| Shock | 7 | 3.32 | 3.32 | 11.35 | 1.58 | 0.34 |  |
| Suicidal ideation | 29 | 3.26 | 3.25 | 45.23 | 2.26 | 1.07 |  |
| Respiratory disorder | 9 | 3.17 | 3.16 | 13.32 | 1.64 | 0.47 |  |
| Urinary incontinence | 9 | 3.11 | 3.11 | 12.89 | 1.62 | 0.45 |  |
| Altered state of consciousness | 6 | 3.10 | 3.09 | 8.51 | 1.39 | 0.16 |  |
| Acute kidney injury | 58 | 3.09 | 3.07 | 81.24 | 2.37 | 1.19 |  |
| Blood creatine phosphokinase increased | 9 | 2.99 | 2.99 | 11.89 | 1.55 | 0.40 |  |
| Dystonia | 6 | 2.99 | 2.98 | 7.92 | 1.34 | 0.13 |  |
| Completed suicide | 25 | 2.93 | 2.92 | 31.55 | 1.97 | 0.87 |  |
| Withdrawal syndrome | 11 | 2.92 | 2.91 | 13.83 | 1.61 | 0.49 |  |
| Fear | 8 | 2.79 | 2.78 | 9.15 | 1.39 | 0.25 |  |
| Cardio-respiratory arrest | 12 | 2.78 | 2.78 | 13.63 | 1.57 | 0.49 |  |
| Drug ineffective for unapproved indication | 13 | 2.76 | 2.76 | 14.60 | 1.60 | 0.52 |  |
| Depressed mood | 13 | 2.65 | 2.65 | 13.34 | 1.54 | 0.47 |  |
| Aggression | 13 | 2.56 | 2.56 | 12.37 | 1.49 | 0.43 |  |
| Drug interaction | 39 | 2.55 | 2.54 | 36.55 | 1.85 | 0.83 |  |
| Off label use | 170 | 2.52 | 2.48 | 151.68 | 2.13 | 1.07 |  |
| Disorientation | 10 | 2.47 | 2.47 | 8.76 | 1.33 | 0.25 |  |
| Intentional product misuse | 27 | 2.47 | 2.46 | 23.53 | 1.69 | 0.68 |  |
| Renal impairment | 19 | 2.46 | 2.45 | 16.35 | 1.56 | 0.55 |  |
| Hypotension | 47 | 2.45 | 2.44 | 40.01 | 1.83 | 0.82 |  |
| Tachycardia | 20 | 2.35 | 2.34 | 15.40 | 1.51 | 0.51 |  |
| Abnormal behaviour | 9 | 2.14 | 2.14 | 5.47 | 1.11 | 0.03 |  |
| Suicide attempt | 12 | 2.05 | 2.05 | 6.48 | 1.16 | 0.12 |  |
| Injury | 15 | 2.05 | 2.04 | 8.00 | 1.23 | 0.22 |  |
