# Supplementary material for: Comparing the adverse effects of ketamine and esketamine between genders using FAERS data
Source: Front Pharmacol. 2024 Jul 12;15:1329436. doi: 10.3389/fphar.2024.1329436 (PMC11272469; doi:10.3389/fphar.2024.1329436)
Supplement: Supplementary file 2 [file DataSheet1.docx]

***Supplementary File 1***

***Comparing the adverse effects of* ketamine *and esketamine between genders using FAERS Data***

## (1) Reporting odds ratio (ROR) method

| **Method** | **Formula** | **Threshold** |
| --- | --- | --- |
| ROR | $ROR=\frac{(a/c)}{(b/d)}=\frac{ad}{bc}$  $SE(lnROR)=\sqrt{(\frac{1}{a}+\frac{1}{b}+\frac{1}{c}+\frac{1}{d})}$  $95\%CI=e^{ln(ROR)\pm1.96\sqrt{(\frac{1}{a}+\frac{1}{b}+\frac{1}{c}+\frac{1}{d})}}$ | When ROR >1 and 95%CI >1, it prompts to generate one signal. |

**References:**

A Real-World Disproportionality Analysis of Olaparib: Data Mining of the Public Version of FDA Adverse Event Reporting System

## (2) Medicines and healthcare products regulatory agency (MHRA) and Proportional reporting ratio (PRR) method

| **Method** | **Formula** | **Threshold** |
| --- | --- | --- |
| MHRA  PRR | $\mathrm{PRR}＝\frac{a/(a+b)}{c/(c+d)}$  ① When $(a+b+c+d)$＜ 40, and T_min_ ＜ 5, use the following formula  $\chi2 =\frac{{(\left\vert ad-bc \right\vert-\frac{a+b+c+d}{2})}^{2}(a+b+c+d)}{( a+b)(a+c)(c+d)(b+d)}$  ② When $\left( a+b+c+d \right)\geq$ 40, and T_min_ ≥ 5, use the following formula  $\chi2 =\frac{{(ad-bc)}^{2}(a+b+c+d)}{( a+b)(a+c)(c+d)(b+d)}$ | When a≥3, PRR≥2 and $\chi2\geq$4, it prompts to generate one signal. |

**References:**

A comparison of measures of disproportionality for signal

detection in spontaneous reporting systems for adverse drug reactions

## (3) Bayesian confidence propagation neural network (BCPNN)

| **Method** | **Formula** | **Threshold** |
| --- | --- | --- |
| BCPNN | IC=${log}_{2}\frac{p(x,y)}{p(x)p(y)}={log}_{2}\frac{a(a+b+c+d)}{(a+b)(a+c)}$  E(IC)=${log}_{2}\frac{(a+\gamma11)(a+b+c+d+\alpha)(a+b+c+d+\beta)}{（a+b+c+d+\gamma）(a+b+\alpha1)(a+c+\beta1)}$  V(IC)=$\frac{1}{{(ln2)}^{2}}\{\left[ \frac{\left( a+b+c+d \right)-a+\gamma-\gamma11}{\left( a+\gamma11 \right)\left( 1+a+b+c+d+\gamma\right)} \right]+\left[ \frac{\left( a+b+c+d \right)-\left( a+b \right)+\alpha-\alpha1}{\left( a+b+\alpha1 \right)\left( 1+a+b+c+d+\alpha\right)} \right]+\left[ \frac{\left( a+b+c+d \right)-\left( a+c \right)+\beta-\beta1}{\left( a+c+\beta1 \right)\left( 1+a+b+c+d+\beta\right)} \right]\}$  $\gamma=\gamma11\frac{(a+b+c+d+\alpha)(a+b+c+d+\beta)}{(a+b+\alpha1)(a+c+\beta1)}$  *IC-2SD=E(IC)-2*$\sqrt{V(IC)}$  $其中\alpha1=\beta1=1；\alpha=\beta=2；\gamma11=1$ | When IC-2SD ＞0, it prompts to generate one signal. |

**References:**

A Real-World Disproportionality Analysis of Olaparib: Data Mining of the Public Version of FDA Adverse Event Reporting System

## (4) Multi-item gamma Poisson shrinker (MGPS) method

| **Method** | **Formula** | **Threshold** |
| --- | --- | --- |
| MGPS | $\mathrm{EBGM}＝\frac{a(a+b+c+d)}{(a+c)(a+b)}$  $95\%CI=e^{ln(EBGM)\pm1.96\sqrt{(\frac{1}{a}+\frac{1}{b}+\frac{1}{c}+\frac{1}{d})}}$ | When EBGM05>2, it prompts to generate one signal. |

**References:**

A Real-World Disproportionality Analysis of Olaparib: Data Mining of the Public Version of

FDA Adverse Event Reporting System
